# Supplementary material for: A unique intracellular tyrosine in neuroligin-1 regulates AMPA receptor recruitment during synapse differentiation and potentiation
Source: Nat Commun. 2018 Sep 28;9:3979. doi: 10.1038/s41467-018-06220-2 (PMC6162332; doi:10.1038/s41467-018-06220-2)
Supplement: Supplementary file 1 — Supplementary Information [file 41467_2018_6220_MOESM1_ESM.pdf]

A unique intracellular tyrosine in neuroligin-1 regulates AMPA receptor recruitment during synapse differentiation and potentiation.

Letellier et al.

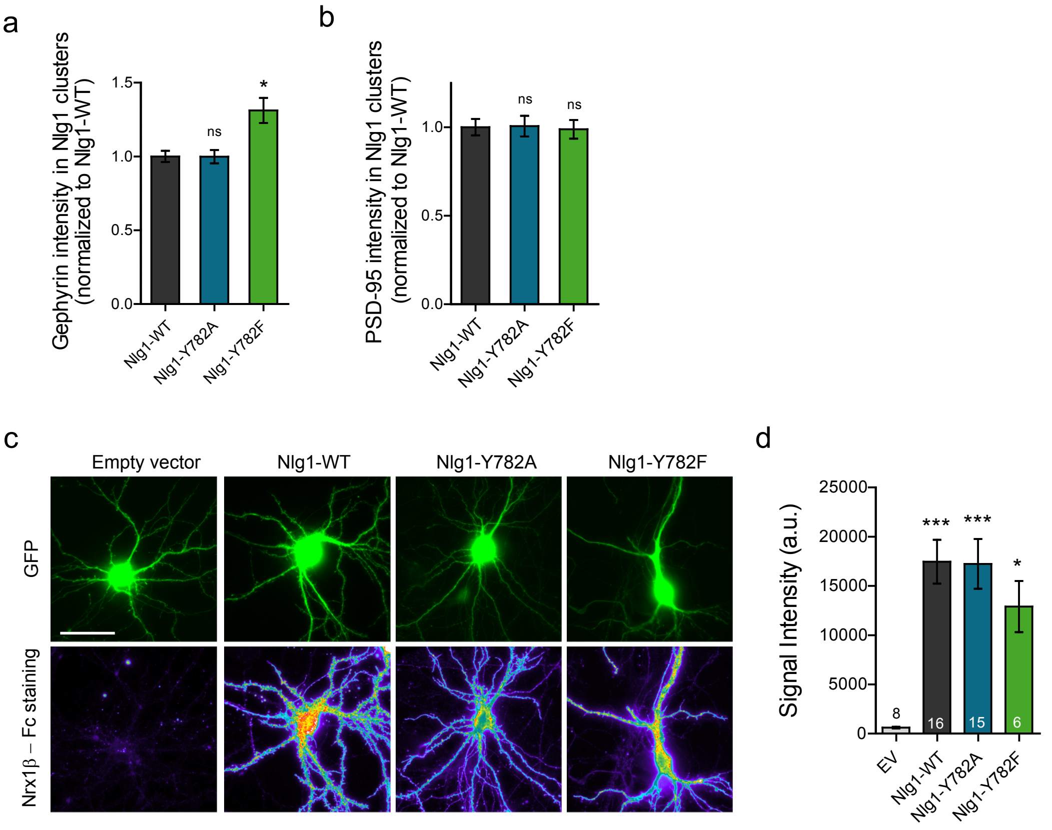

Supplementary Figure 1. Immunofluorescence intensity of gephyrin and PSD-95 in Nlg1 clusters, and binding of Nlg1 mutants to Nrx1β

Average fluorescence intensity of immunostained gephyrin **(a)** and PSD-95 **(b)** in isolated Nlg1 clusters, normalized to the control condition. (Kruskal-Wallis test followed by Dunn's multiple comparison test, *P < 0.05). **(c)** Live labeling of neurons expressing GFP (green) + EV, Nlg1-WT, Nlg1-Y782A, or Nlg1-Y782F with soluble Nrx1β-Fc, followed by fixation and immunostaining with Alexa647-conjugated anti-Fc antibody (pseudocolor). Scale bar, 50 µm. **(d)** The graph represents the average fluorescence intensity of immunostained Nrx1β-Fc in the four conditions, in arbitrary units. Data represent mean ± SEM and were compared by one-way ANOVA followed by Tukey's multiple comparison test (*P < 0.05, ***P < 0.001).

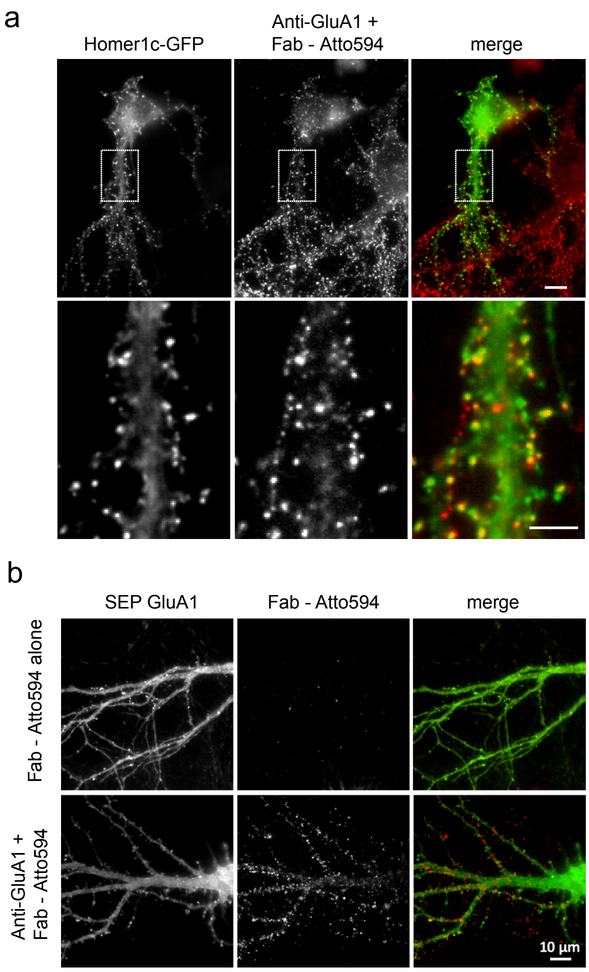

Supplementary Figure 2. Specificity of GluA1 immunostaining for single molecule tracking

(a) Epifluorescence images showing neurons expressing Homer-1c-GFP (green) immunostained for endogenous AMPARs at the cell surface using a mix of rabbit anti-GluA1 and Atto594-conjugated anti-rabbit Fab (red). Note the synaptic localization of endogenous GluA1 at Homer-1c puncta. Scale bars, 10 µm (upper panels) and 5 µm (bottom panels). **(b)** Epifluorescence images showing neurons expressing SEP-GluA1 (green) immunostained with the anti-GluA1/Fab mix (red). The fluorescence signal is reduced when the primary antibody is omitted (upper panels), revealing labeling specificity for GluA1.

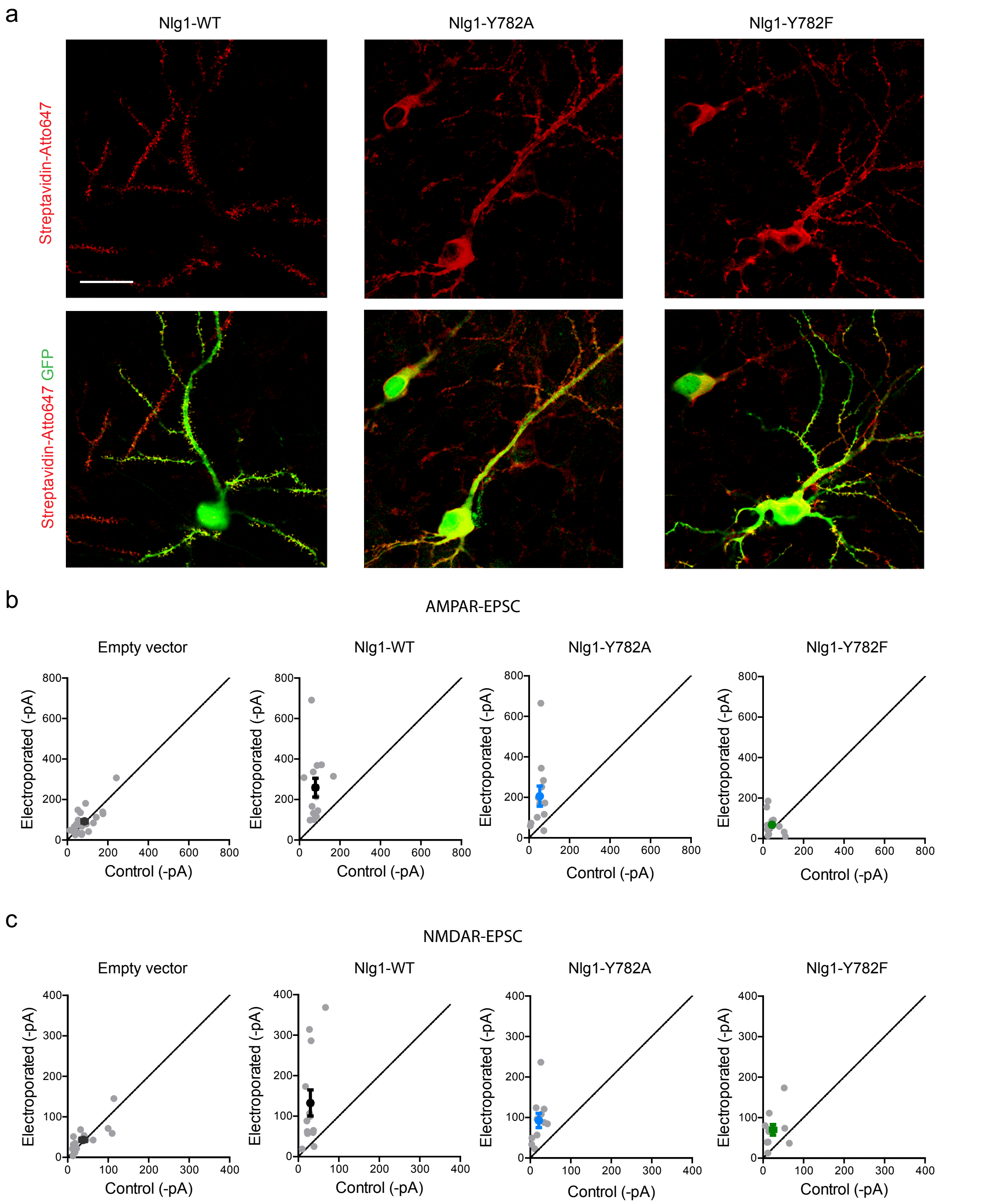

Supplementary Figure 3. Co-expression of GFP and Nlg1-WT, -Y782A or -Y782F in electroporated CA1 neurons, and EPSC amplitudes in CA1 neurons expressing Nlg1 tyrosine point mutants

(a) Confocal sections showing the co-expression of GFP (green) and biotinylated AP-Nlg1 constructs stained with streptavidin-Atto647 (red) in electroporated CA1 neurons, for Nlg1-WT and the two point mutants. Scale bar, 30 µm. **(b,c)** Scatter plots of AMPAR- and NMDAR-mediated EPSC amplitudes, respectively, showing the individual conditions summarized in Fig. 4c and 4d. CA1 neurons electroporated with EV, Nlg1-WT, Nlg1-Y782A or Nlg1-Y782F (y-axis) are compared to paired non-electroporated neurons (control, x-axis). Data represent mean ± SEM.

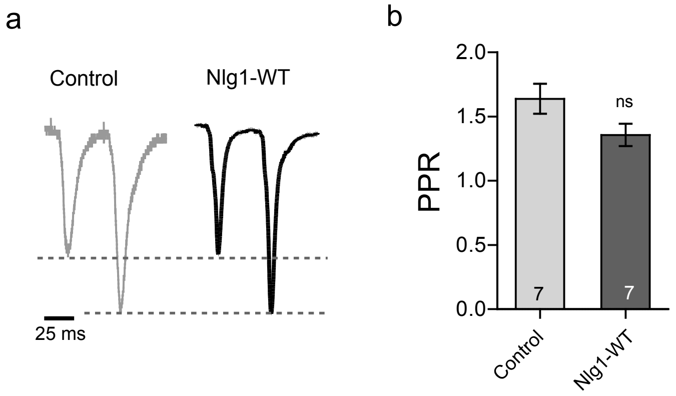

Supplementary Figure 4. The paired pulse ratio (PPR) is not affected by Nlg1-WT expression in CA1 neurons

(a) Average EPSC traces in response to paired stimuli (50 ms interstimulus interval) recorded from a neuron expressing Nlg1-WT (in black) and a neighboring unelectroporated neuron (control, in grey). **(b)** Average PPR for control and Nlg1-WT expressing neurons (Wilcoxon matched-pairs signed rank test, ns: not significant). Data represent mean ± SEM.

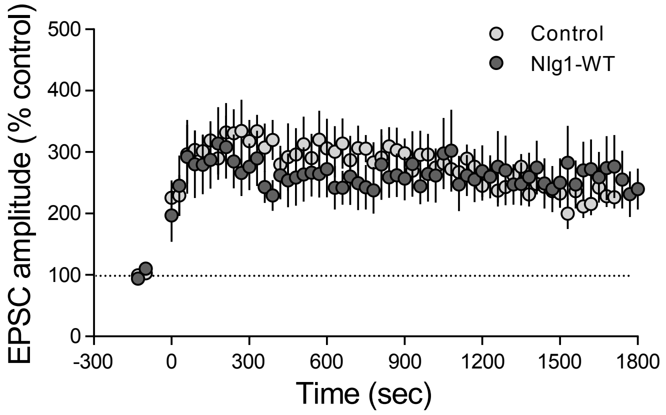

Supplementary Figure 5. CA1 neurons electroporated with Nlg1 shRNA and rescue Nlg1-WT display normal LTP compared to non-electroporated neurons

AMPAR-mediated EPSCs for CA1 neurons from Nlg1 WT mice electroporated with Nlg1 shRNA + Nlg1-WT rescue (black), and for neighboring non-electroporated neurons (control, grey), upon LTP induction at time 0. Data were compared to the control condition (unelectroporated) by Wilcoxon matched-pairs signed rank test (ns: not significant). Data represent mean ± SEM.

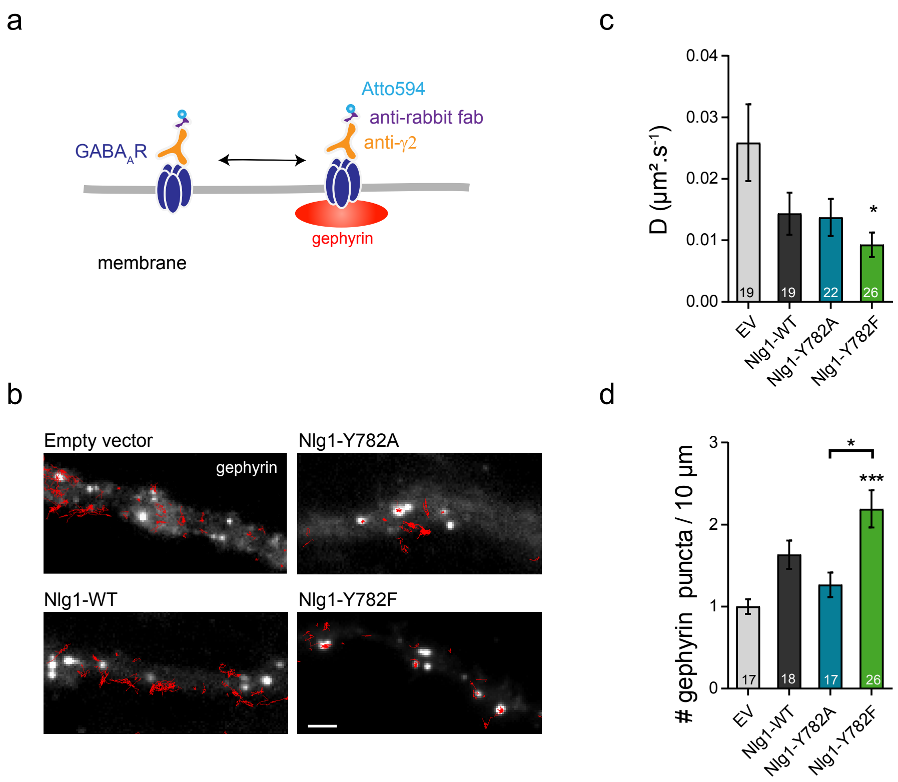

Supplementary Figure 6. Nlg1-Y782F preferentially recruit gephyrin and capture surface diffusing GABA$_A$ receptors

(a) Neurons were live labelled for endogenous GABA$_A$ receptors with anti-γ 2 antibody mixed with Atto594-conjugated anti-rabbit Fab. Inhibitory post-synapses were detected with Gephyrin-Venus. **(b)** Representative trajectories of individual GABA$_A$ receptors at the neuronal surface (red) in 10 DIV neurons expressing EV, Nlg1-WT, -Y782A or -Y782F along with Gephyrin-Venus (white). Scale bar, 2 µm. **(c)** The median diffusion coefficient per cell was averaged for each condition (4 independent experiments). The number of cells examined is given in the columns. **(d)** Effect of the Nlg1 mutations on the number of Gephyrin-Venus puncta per unit length of dendrite. Data in graphs (c) and (d) represent mean ± SEM and were compared by non parametric ANOVA (Kruskal-Wallis) followed by Dunn's multiple comparison test (*P < 0.05, ***P < 0.001).

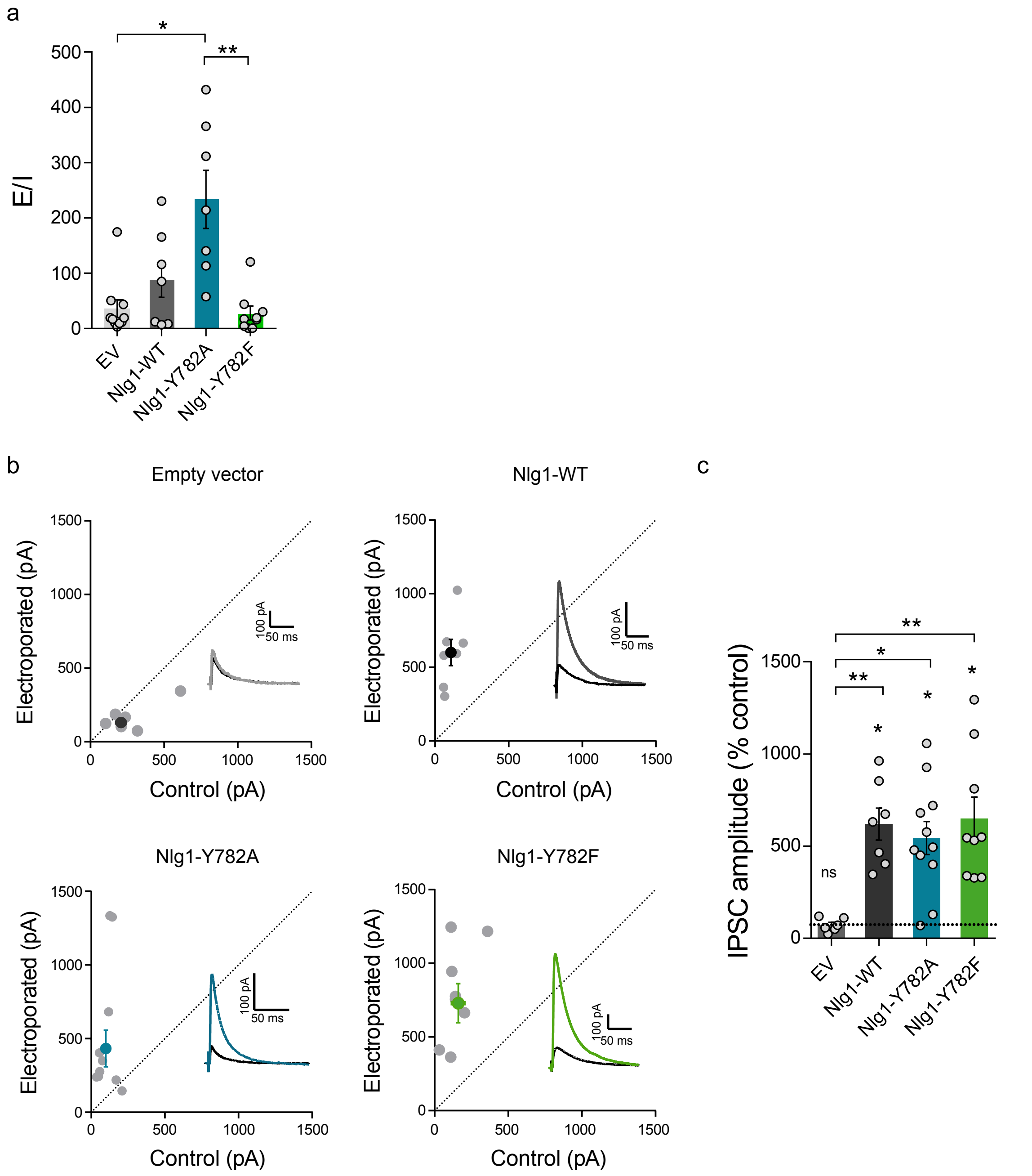

Supplementary Figure 7. Expression of both Nlg1-Y782A and Nlg1-Y782F in CA1 neurons from Nlg1 KO mice enhances IPSCs to the same extent as Nlg1-WT

(a) Graph showing the paired ratio between the frequency of mEPSCs and mIPSCs measured cell by cell, in cultured hippocampal neurons expressing EV, Nlg1-WT, Nlg1-Y782A or Nlg1-Y782F. **(b)** Scatter plots of the amplitude of IPSCs evoked in CA1 neurons electroporated with EV, Nlg1-WT, Nlg1-Y782A or Nlg1-Y782F and compared to neighbor unelectroporated neurons (control). Example traces are shown in color for each condition (control trace are in black). **(c)** Summary of IPSC amplitudes normalized to the control neuron for the same conditions (number of pairs indicated within bars, from 3 independent experiments). Data represent mean ± SEM. Data in graph (a) were compared by a Kruskal-Wallis test followed by Dunn's multiple comparison test (*P < 0.05, **P < 0.01). Data in graph (c) were compared to the control condition by Wilcoxon matched-pairs signed rank test, and between themselves using one-way ANOVA followed by Tukey's multiple comparison (ns: not significant, *P < 0.05, **P < 0.01). Data represent mean ± SEM.

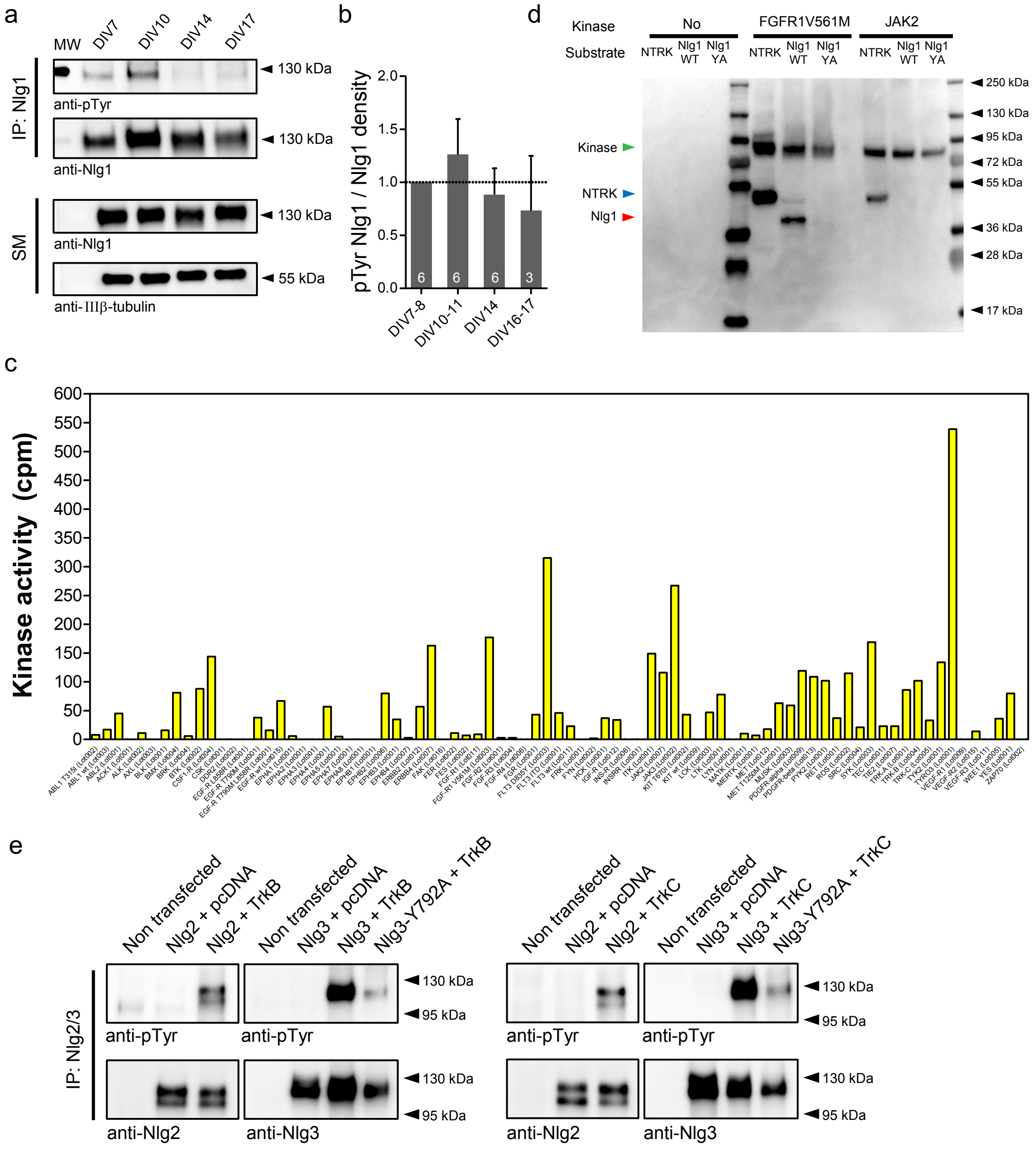

Supplementary Figure 8. Regulation of Nlg phosphorylation across development and by specific tyrosine kinases

(a) Tyrosine phosphorylation of Nlg1 during development. Cortical neurons were cultured for 7-8, 10-11, 14, or 16-17 DIV, then lysed and protein extracts were immuno-precipitated with Nlg1 antibodies, and run on a polyacrylamide gel. pTyr and Nlg1 immunoblots are shown for the immunoprecipitation (IP), as well as Nlg1 and IIIβ-tubulin immunoblots for the starting material (SM). **(b)** pTyr signals normalized to Nlg1 levels for the different time points. The number of individual experiments is indicated. **(c)** Screen of tyrosine kinases phosphorylating Nlg1. Biotinylated Nlg1 peptides comprising the 16 aa gephyrin-binding motif were immobilized on streptavidin-coated wells and incubated with 81 different tyrosine kinases in the presence of radioactive ATP. Kinase activity values corrected for peptide background are given in counts per million (cpm), with sample peptide at one concentration (1 μM). **(d)** In vitro kinase assay showing the phosphorylation of GST-Nlg1 by constitutively active purified FGFR1 (FGFR1V561M), but not by JAK2. The red arrowhead indicates the major band of GST-Nlg1, the blue arrowhead the position of the positive kinase substrate NTRK, and the green arrowhead the position of auto-phosphorylated tyrosine kinases FGFR1 and JAK2. **(e)** Phosphorylation of Nlg2 and Nlg3 by TrkB/C in COS-7 cells. COS cells expressing Nlg2, Nlg3, or Nlg3-Y792A,with or without TrkB or TrkC, were lysed and protein extracts were immuno-precipitated with Nlg2 or Nlg3 antibodies and immunoblotted for pTyr, Nlg2, or Nlg3.

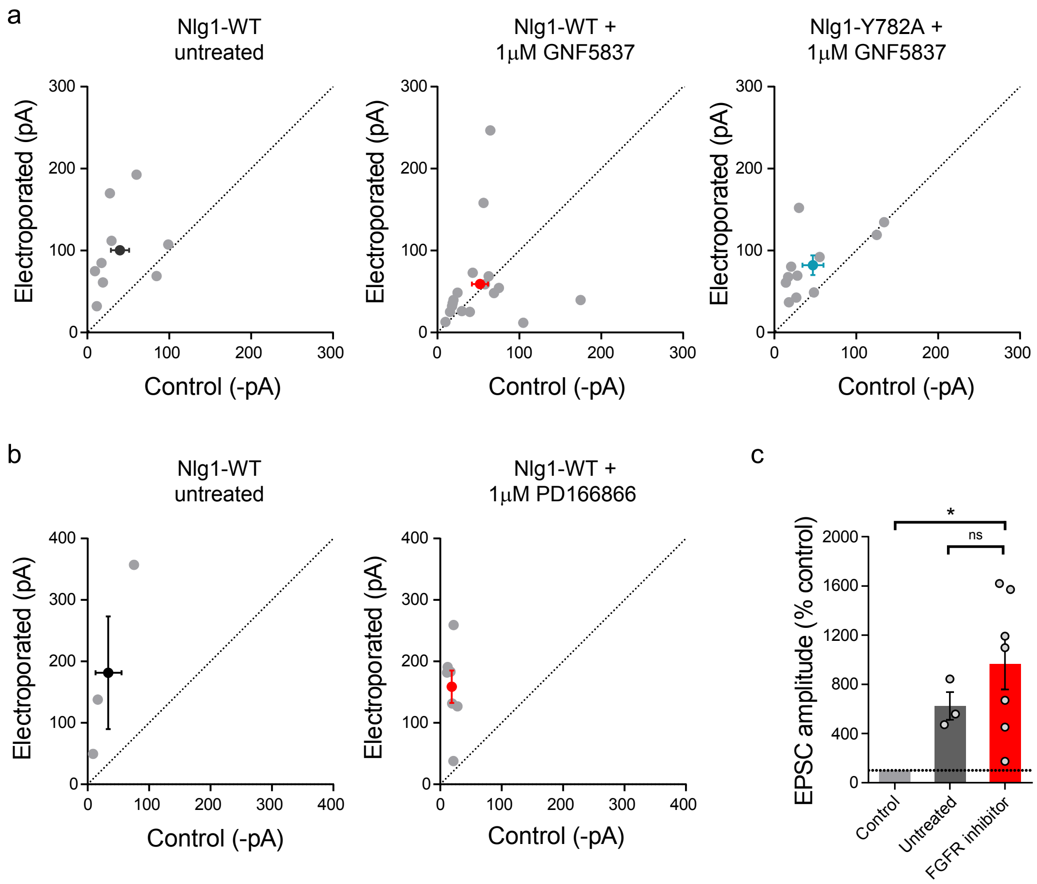

Supplementary Figure 9. Enhancement of AMPAR-mediated EPSCs in CA1 cells from Nlg1 KO mice electroporated with Nlg1-WT is blocked with a pan-Trk inhibitor but not with an FGFR1 inhibitor

(a) Scatter plots of AMPAR-EPSC amplitudes showing the individual conditions summarized in Figs. 8h, i. CA1 neurons electroporated with Nlg1-WT or Nlg1-Y782A and treated or not with a panTrk inhibitor (GNF5837, 1µM) are compared to neighbor unelectroporated neurons (control). **(b)** Scatter plots of AMPARs-EPSCs amplitude for CA1 neurons electroporated with Nlg1-WT and treated or not with FGFR1 inhibitor (PD166866, 1µM). **(c)** Summary of EPSC amplitudes normalized to the control neuron for the same conditions as in (b) (number of pairs indicated within bars, from 1 experiment). Data in graph (c) were compared to the control condition or between themselves by Wilcoxon matched-pairs signed rank test (ns: not significant, $^*P < 0.05$). Data represent mean ± SEM.

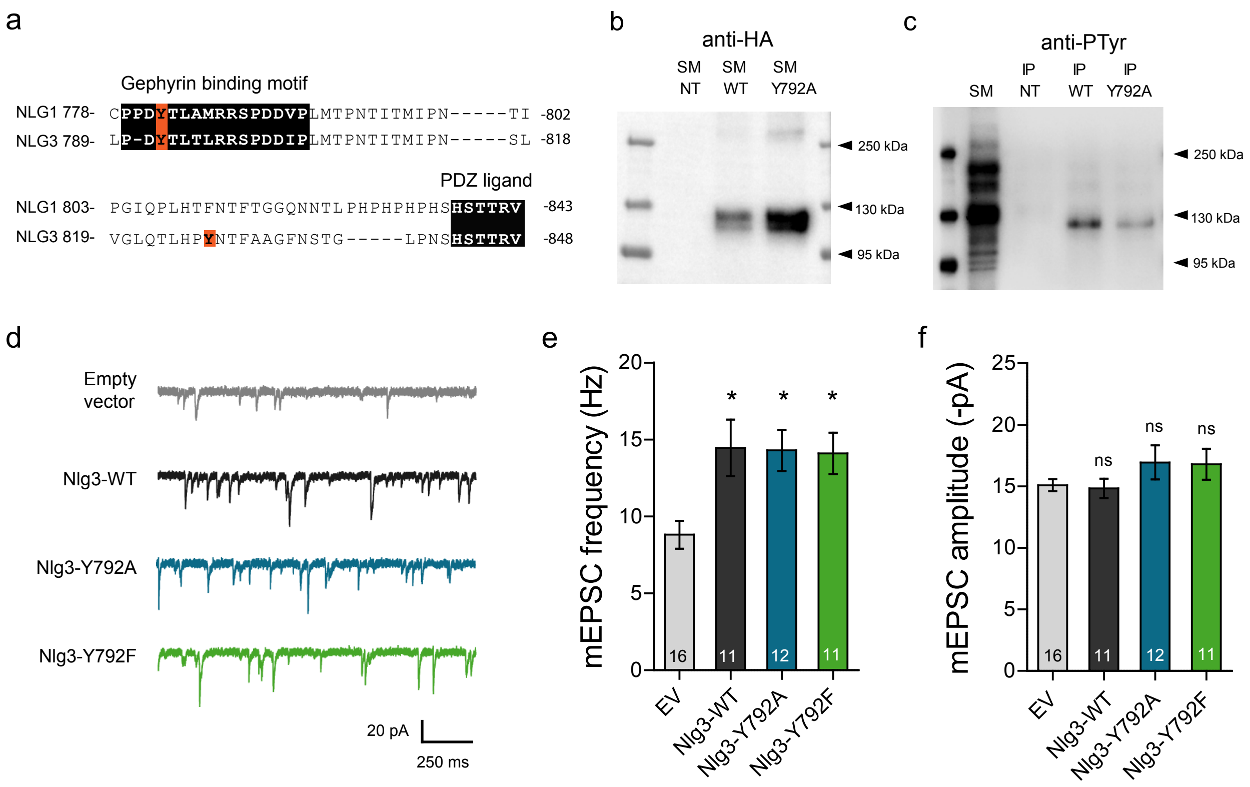

Supplementary Figure 10. Nlg3 is tyrosine phosphorylated but overexpression of Nlg3-Y792A and Nlg3-Y792F mutants both increase mEPSC frequency in dissociated hippocampal neurons

(a) Sequence alignment of the C-terminal domain of rat Nlg1 and Nlg3 highlighting conserved gephyrin- and PDZ domain-binding motifs (black boxes). Tyrosine residues are indicated in orange. **(b)** Anti-HA immunoblot performed on extracts from COS-7 cells either not transfected (NT), or transfected with Nlg3-WT or Nlg3-Y792A (SM: starting material). **(c)** Corresponding pTyr immunoblot after immunoprecipitation of HA-Nlg3 with an anti-HA antibody. **(d)** Representative traces of mEPSC recordings from 14-15 DIV neurons expressing EV, Nlg1-WT, -Y792A or -Y792F clamped at -70 mV in the presence of TTX and bicuculline. **(e, f)** Mean mEPSC frequencies and amplitudes, respectively, for each condition (number of cells indicated within bars, from 4 independent experiments). Data represent mean ± SEM and were compared by one-way ANOVA followed by Tukey's multiple comparison test (ns: not significant, *$P < 0.05$).

Figure 8c

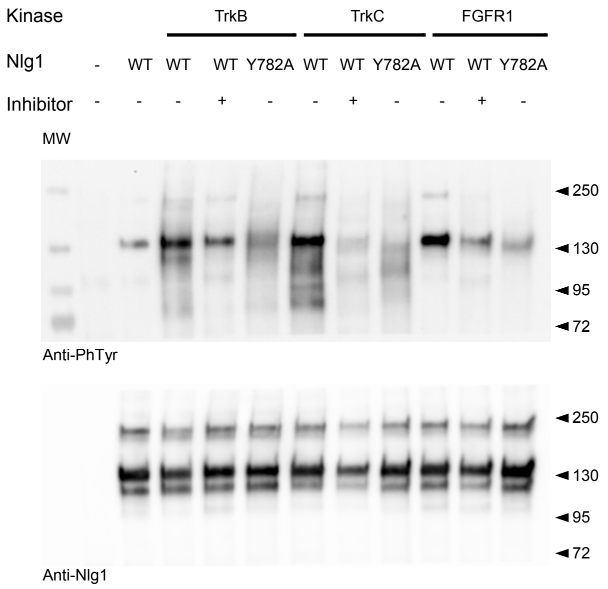

Figure 8e

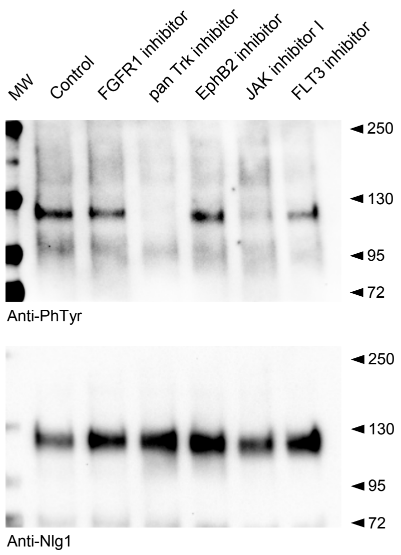

Figure 8f

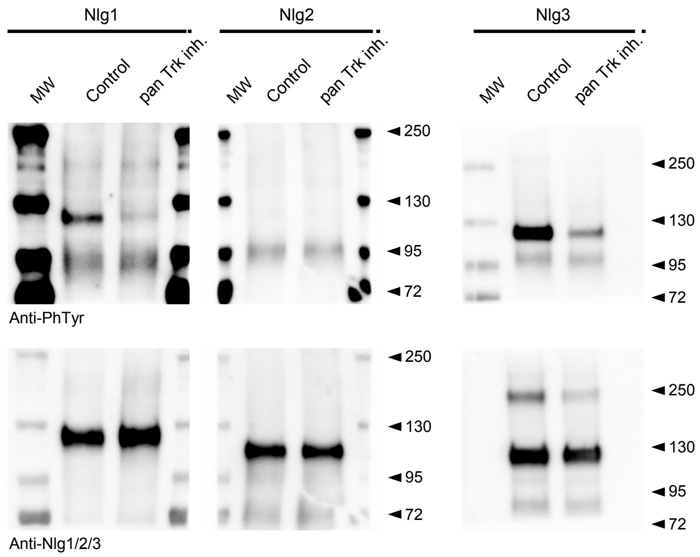

Supplementary Figure 11. Uncropped scans of immunoblots shown in Figure 8
